# Hepatitis C virus core protein substitutions affect the response to pegylated-interferon and ribavirin therapy

**DOI:** 10.1186/1471-2164-15-S2-P5

**Published:** 2014-04-02

**Authors:** Fatimah S Alhamlan, Mohammed N Al-Ahdal, Nisreen Z Khalaf, Ayman A Abdo, Faisal Sanai, Hamad I Al-Ashgar, Ahmed A Al-Qahtani

**Affiliations:** 1 Department of Infection and Immunity, King Faisal Specialist Hospital and Research Center, Riyadh, Saudi Arabia; 2 Department of Pathology and Laboratory Medicine, King Faisal Specialist Hospital and Research Center, Riyadh, Saudi Arabia; 3 Department of Microbiology and Immunology, College of Medicine, Alfaisal University, Riyadh, Saudi Arabia; 4 Liver Disease Research Center, King Saud University, Riyadh, Saudi Arabia; 5 Section of Gastroenterology, Department of Medicine, College of Medicine, King Saud University, Riyadh, Saudi Arabia; 6 Hepatobiliary Sciences and Liver Transplantation, King Abdulaziz Medical City, Riyadh, Saudi Arabia; 7 Department of Medicine, King Faisal Specialist Hospital & Research Center, Riyadh, Saudi Arabia

## Background

Hepatitis C virus (HCV) shows remarkable genetic diversity, which contributes to its high persistence and varied susceptibilities to antiviral treatments. Previous studies have reported that the substitution of amino acids in the HCV-1b core region at positions 70 (Arg70 to Gln70) and/or 91 (Lue91 to Met91) is associated with a poor response to pegylated- interferon and ribavirin (PEG-IFN/RBV) therapy [1,2]. Because the role of the core protein in HCV infections is unclear in Saudi populations, we aimed in this study to analyze the full-length core protein sequences from Saudi patients.

## Materials and methods

A total of 300 samples were obtained from Saudi patients who went through PEG-IFN/RBV treatment. Samples were divided further to responder and non-responder groups. Direct sequencing was employed, followed by sequence analyses using advanced software.

## Results

Our data showed that there was significant association between core protein mutations, particularly at position 70, and treatment outcome in HCV1b and HCV-4d but not in HCV-1a and HCV-4a clinical samples. In addition, amino acid residue at position 91 was well-conserved among all clinical samples where Cys91 is the dominant amino acid residue. Furthermore, our data reported point mutations at different positions that were flagged as ‘rare’ mutations by negative BLOSUM scores.

## Conclusions

Amino acid substitution pattern differs substantially among HCV sub-genotypes. Such discrepancy needs further investigations. Our finding provides a new insight into HCV among effected Saudi population where the knowledge of HCV polymorphisms is lacking.
